# Development and Application of a Dual-Fluorescent Reporter Assay for Feline Infectious Peritonitis Virus RNA-Dependent RNA Polymerase Inhibitor Screening

**DOI:** 10.3390/ijms27198682

**Published:** 2026-09-28

**Authors:** Ploypailin Semkum, Varanya Lueangaramkul, Supichaya Jiwattanasap, Natjira Mana, Nantawan Phetcharat, Porntippa Lekcharoensuk, Sirin Theerawatanasirikul

**Affiliations:** 1 Department of Microbiology and Immunology, Faculty of Veterinary Medicine, Kasetsart University, Bangkok 10900, Thailand; fvetpls@ku.ac.th (P.S.); natjira.ma@ku.th (N.M.); fvetptn@ku.ac.th (P.L.); 2 Department of Anatomy, Faculty of Veterinary Medicine, Kasetsart University, Bangkok 10900, Thailand; varanya.luea@ku.th; 3 Graduate Program in Animal Health and Biomedical Sciences, Faculty of Veterinary Medicine, Kasetsart University, Bangkok 10900, Thailand; supichaya.jiw@ku.th

**Keywords:** feline infectious peritonitis virus, RNA-dependent RNA polymerase (RdRp), cell-based, antiviral compounds, screening platform

## Abstract

Feline infectious peritonitis virus (FIPV) causes a fatal disease in cats that remains challenging to treat due to the absence of effective vaccines and limited antiviral therapies. Given its central role in viral replication and transcription, RNA-dependent RNA polymerase (RdRp) represents a highly promising and strategic target for antiviral drug development. To enable live-cell monitoring of RdRp activity without external substrates or cell disruption, we developed a biologically validated, cell-based dual-fluorescent reporter assay using green fluorescent protein and red fluorescent protein to evaluate RdRp-dependent reporter activity. Following validation with the well-characterized RdRp inhibitor ribavirin, the dual-reporter platform achieved a Z-factor of 0.54 and a Z′-factor of 0.61. The system was further utilized to assess the antiviral potential of fifteen alkaloid compounds. Among compounds evaluated, lycorine and lycorine hydrochloride exhibited potent inhibition of RdRp-dependent reporter activity and yielded IC_50_ values of 2.83 ± 0.45 μM and 1.58 ± 0.20 μM, respectively. Further evaluation revealed that both compounds significantly reduced FIPV replication in infected cell. These findings identify lycorine and lycorine hydrochloride as promising anti-FIPV candidates and support further investigation of lycorine-class alkaloids as anti-coronaviral scaffolds. Together, the validated cell-based screening platform and whole-virus antiviral activity provide a foundation for advancing RdRp-targeted antiviral discovery against FIPV.

## 1. Introduction

Feline infectious peritonitis (FIP) is a progressive, often fatal immune-mediated disease caused by pathogenic variants of feline coronavirus (FCoV), which exists as two biotypes: feline enteric coronavirus (FECV) and feline infectious peritonitis virus (FIPV) [1,2]. FCoV is highly prevalent in cats, with serum antibody seroprevalence ranging from under 20% in single-cat or stray populations to as high as 80–100% in multi-cat and cattery environments [3], of which approximately 5–12% develop FIP [4]. FIPV arises when FECV acquired within-host mutations that enable replication in monocytes and macrophages and drive fatal systemic disease [1,2,3]. The recent emergence of a recombinant FCoV–canine coronavirus (FCoV-23) behind a rapidly spreading FIP outbreak in Cyprus further underscores the evolving epidemiological threat and cross-species recombination potential of FCoV [5,6]. Despite recent antiviral advances, FIP remains a major clinical challenge, highlighting the continued need for effective antiviral strategies [3].

FCoV belongs to the family *Coronaviridae*, genus *Alphacoronavirus* [1,2,7]. Its approximately 29 kb positive-sense, single-stranded RNA genome encodes four structural proteins—spike (S), envelope (E), membrane (M), and nucleocapsid (N) proteins—together with several accessory proteins and a replicase polyprotein that is processed into multiple non-structural proteins (nsps) essential for viral replication [1,2,7]. Central to viral RNA synthesis is the RNA-dependent RNA polymerase (RdRp; nsp12), which assembles with its essential cofactors nsp7 and nsp8 into a highly processive replication complex that synthesized the negative-sense RNA intermediates used as templates for genomic and subgenomic RNA production [8,9,10]. Structural studies have shown that the FCoV nsp7–nsp8 complex is highly conserved with its counterparts in other alpha- and betacoronaviruses [9], and as one of the most conserved proteins across RNA virus families [10], RdRp represents an attractive target for broad-spectrum antiviral development. Consistent with this, RdRp inhibitors have shown antiviral efficacy against Middle East respiratory syndrome coronavirus (MERS-CoV) and severe acute respiratory syndrome coronavirus 2 (SARS-CoV-2) [11,12], as well as against other RNA viruses, including foot-and-mouth disease virus (FMDV) [13], Zika virus (ZIKV) [14], and FIPV [3,15,16], supporting RdRp as a promising therapeutic target for FCoV infection.

Antiviral drug discovery increasingly relies on computational approaches, such as structure-based virtual screening and molecular docking, to identify compounds targeting viral proteins. However, activity predicted computationally or measured in biochemical enzyme assays does not always translate into antiviral activity in living cells, as these approaches do not fully capture cellular factors such as membrane permeability, intracellular metabolism, compound stability, and cytotoxicity [17]. As a result, compounds with potent enzymatic inhibition may show limited antiviral activity in cell-based systems. To bridge this gap, several cell-based platforms have been developed, including luciferase reporter assays [18,19], fluorescent reporter systems [20,21], stable cell lines expressing viral replication proteins [22], and more recently, FCoV replicons and recombinant reporter-tagged viruses [23]. While these systems have significantly advanced coronavirus antiviral research, many require complex genetic manipulation, specialized reagents, or elaborate experimental procedures that limit their scalability and broad adoption. A simple, robust, and quantitative cell-based assays for evaluating FIPV RdRp inhibitors therefore remains an important unmet need.

In this study, we developed and optimized a fluorescence-based dual-reporter assay to quantitatively monitor FIPV RdRp activity in living cells. The assay employs green fluorescent protein (GFP) as an RdRp-dependent reporter and red fluorescent protein (RFP) as an internal control to normalize transfection efficiency and cell abundance, thereby enabling sensitive and quantitative assessment of RdRp-dependent reporter activity. Using this platform, we evaluated established antiviral compounds, including ribavirin, GS-441524, and molnupiravir, together with a panel of alkaloid compounds. The assay identified lycorine and lycorine hydrochloride as potent inhibitors of FIPV RdRp, and their antiviral activities were subsequently validated using infectious virus assays. Collectively, these findings establish the dual-reporter system as a robust and reliable platform for identifying FCoV RdRp inhibitors and provide a practical foundation for future high-throughput screening and the discovery of RdRp-targeted therapeutics against FIP.

## 2. Results

### 2.1. Establishment of the Cell-Based Dual-Reporter and RdRp Complex Proteins (nsp12, nsp7, and nsp8)

**Figure 1:**
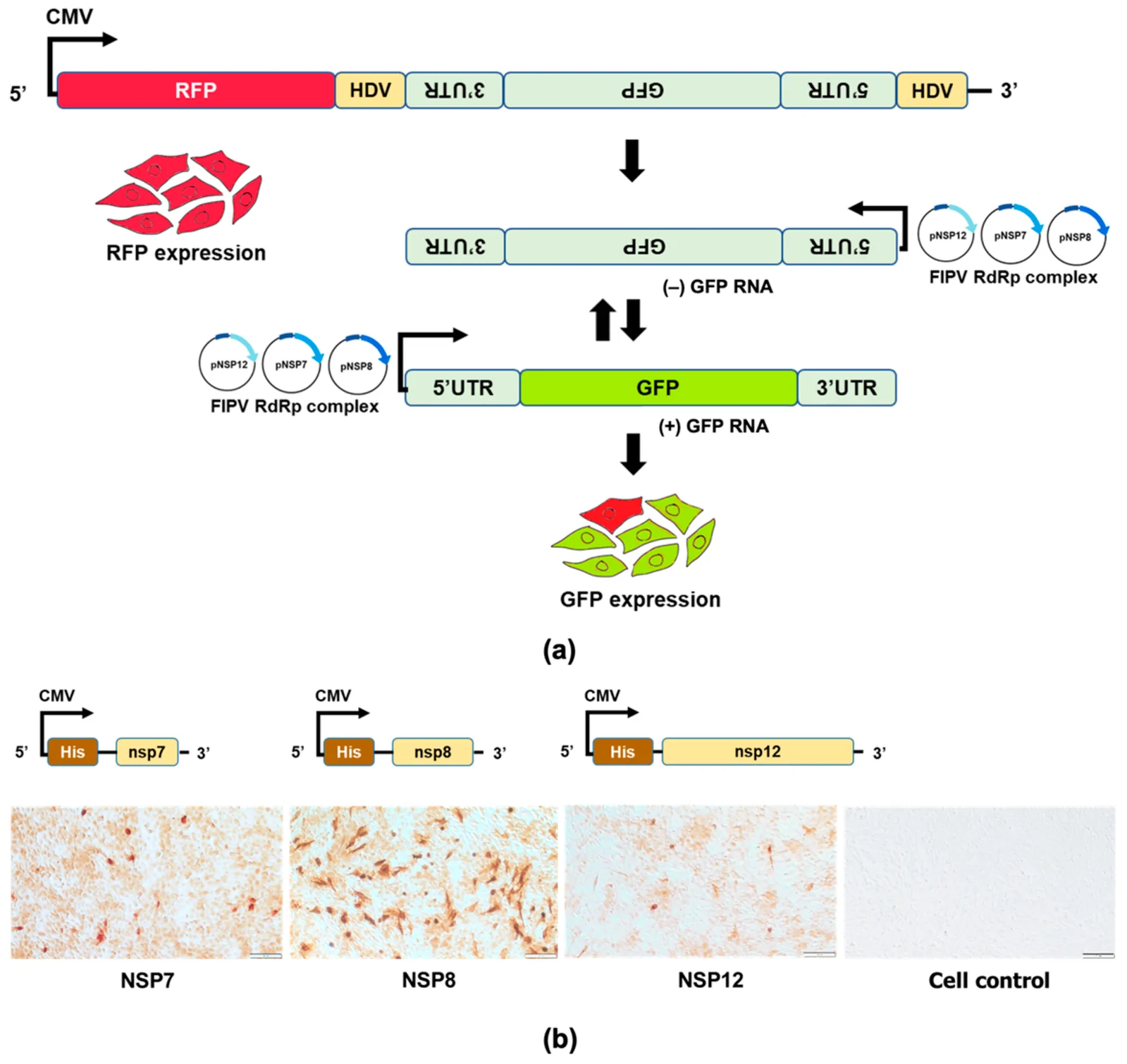
Schematic of dual-reporter cell-based assay system. (**a**) The dual-reporter plasmid reports FIPV RdRp activity through green fluorescence (GFP), with red fluorescence (RFP) as an internal transfection control. Under a CMV promoter, the construct carries the RFP gene in the forward orientation, followed by a GFP cassette flanked by the FIPV 3′- and 5′-UTRs in the reverse orientation. Transcription yields RFP mRNA and a negative-sense FIPV-3′UTR-GFP-5′UTR RNA released by self-cleaving ribozymes at both ends. Co-expressed FIPV nsp7, nsp8, and nsp12 recognize the 5′-UTR and replicate this intermediate into a positive-sense 5′UTR-GFP-3′UTR transcript, enabling GFP expression; FIPV RdRp activity is therefore quantified indirectly from GFP expression. (**b**) Expression of His-tagged FIPV nsp7, nsp8, and nsp12 in BHK-21 cells detected by IPMA; brown spots indicate protein expression.

A cell-based dual-reporter assay was developed to assess FIPV RdRp activity through fluorescence-based readouts. The dual-reporter construct consists of a bicistronic system encoding mCherry (red fluorescent protein; RFP) gene in the sense orientation and the eGFP (green fluorescent protein; GFP) gene in the reverse orientation, under the control of FIPV untranslated regions (UTRs). Specifically, the eGFP gene is flanked by the viral 5′ and 3′ UTRs and positioned between two HDV ribozyme self-cleavage sequences (Figure 1a). Transcription of the dual-reporter RNA is driven by host RNA polymerase II under the CMV promoter. After transcription, HDV ribozyme-mediated self-cleavage generates two RNA species: a positive-sense mCherry mRNA and a negative-sense of 3′UTR-eGFP-5′UTR RNA intermediate. In the presence of FIPV RdRp, the negative-sense RNA is replicated into a positive-sense eGFP transcript, enabling GFP expression. Thus, RFP serves as an internal control for transfection efficiency, whereas GFP fluorescence directly reflects RdRp activity. To express the FIPV RdRp complex in mammalian cells, His-tagged nsp12, nsp7, and nsp8 were transiently expressed following plasmid transfection. Protein expression was confirmed by IPMA, as evidenced by intracellular brown staining in transfected cells (Figure 1b).

### 2.2. Verification and Optimization of the Cell Based Dual-Reporter System

**Figure 2:**
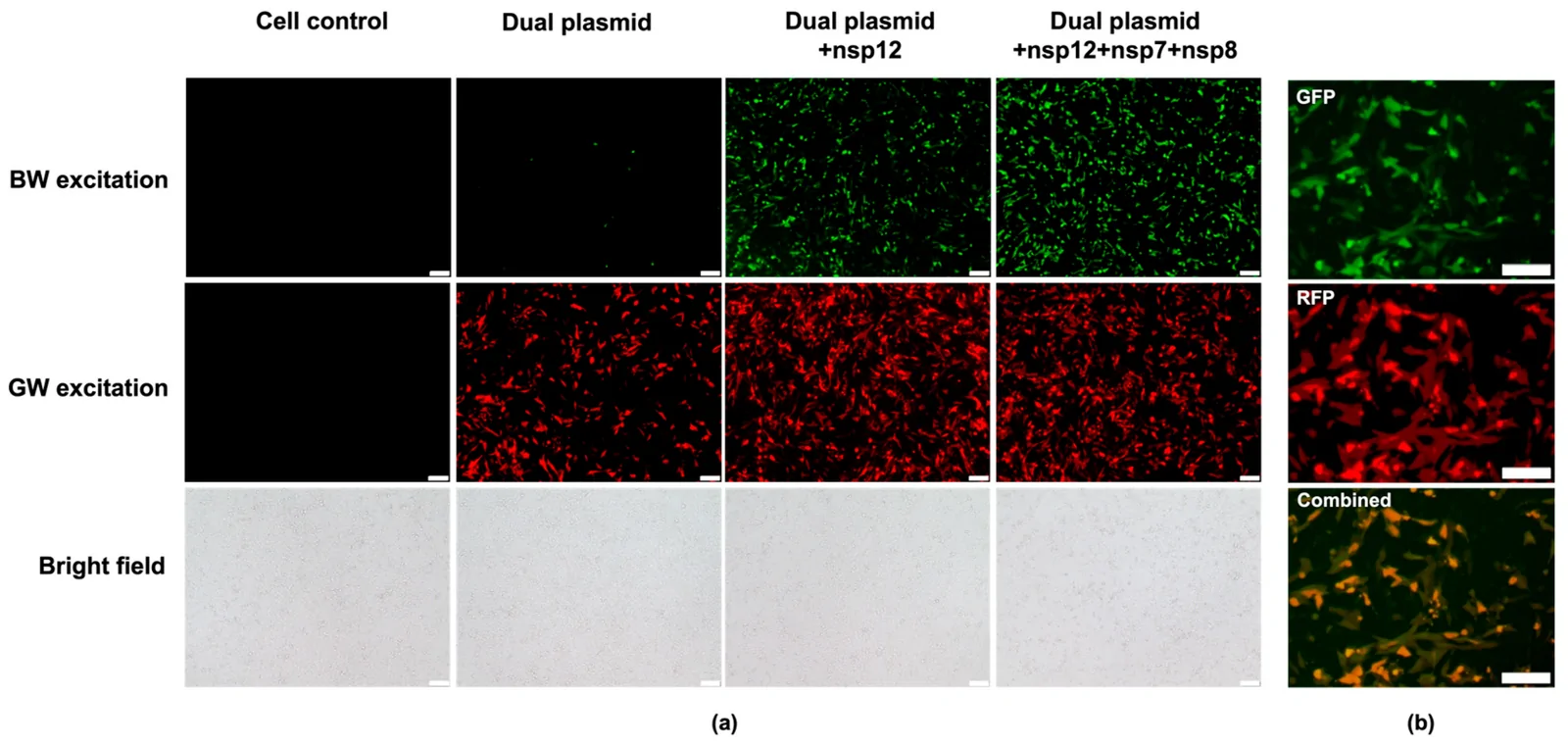
Evaluation of the dual-reporter assay by GFP and RFP fluorescence detection. (**a**) Representative fluorescence images of BHK-21 cells at 24 hpt. GFP reports FIPV RdRp-dependent reporter activity in cells transfected with the dual-reporter plasmid alone, with nsp12 alone, or with the complete nsp12/nsp7/nsp8 complex. RFP served as an internal transfection control, and bright-field images show cell morphology. (**b**) Merged GFP and RFP images; cells expressing the complete nsp12/nsp7/nsp8 complex display both signals, appearing orange. Scale bars, 100 µm.

Previous studies have shown that coronavirus RdRp activity depends on the coordinated interaction of nsp12 with its essential cofactors, nsp7 and nsp8, which together form the replication–transcription complex (RTC) [8,18,22,24,25,26,27]. Based on this, the cell-based dual-reporter assay was optimized by co-transfecting the dual-reporter plasmid with different ratios of FIPV nsp12 expression plasmid, alone or in combination with the nsp7 and nsp8 expression plasmids, to determine the optimal conditions for RdRp-mediated reporter activation. At 24 h post-transfection (hpt), GFP fluorescence was evaluated as a readout of FIPV RdRp activity, whereas RFP fluorescence served as an internal transfection control. Representative fluorescence images showed that cells co-transfected with the dual-reporter plasmid and the complete RdRp complex (nsp12, nsp7, and nsp8) exhibited stronger GFP fluorescence than cells co-transfected with the dual-reporter plasmid and nsp12 alone (Figure 2). In contrast, cells transfected with the dual-reporter plasmid alone displayed minimal GFP fluorescence, indicating the absence of RdRp-dependent reporter activation (Figure 2a). Merged fluorescence images confirmed the co-expression of GFP and RFP in cells co-transfected with the complete RdRp complex (Figure 2b).

Subsequent optimization of plasmid ratios showed that GFP fluorescence increased in a dose-dependent manner with increasing amounts of the nsp12 expression plasmid (Figure S1). Co-transfection of the dual-reporter plasmid with the complete RdRp complex at a ratio of 1:5:1:1 (dual-reporter:nsp12:nsp7:nsp8) produced the highest GFP reporter activity, whereas co-transfection with nsp12 alone at a ratio of 1:5 (dual-reporter:nsp12) resulted in slightly lower reporter activation. Consistently, GFP signal counts were approximately 5.15- and 6.18-fold higher than the reporter-only control for cells co-transfected with the dual-reporter plasmid plus nsp12 alone and plus the complete RdRp complex (nsp12/nsp7/nsp8), respectively (Figure S1b,c). Both configurations produced reporter activity well above background, and the GFP/RFP ratio was higher for the complete complex than for nsp12 alone (0.99 vs. 0.77; Figure S1c), indicating that the increased GFP signal was not attributable to transfection variation. These results show that nsp12 is sufficient to initiate RdRp-dependent reporter expression, with nsp7 and nsp8 providing a modest further enhancement. Time-course analysis further showed that reporter activity peaked between 24 and 32 hpt and declined after 36–48 hpt, while cell morphology remained normal throughout the observation period (bright-field images, Figure S2). Together, these findings demonstrate that the dual-reporter assay responds to FIPV RdRp activity and that co-expression of nsp12, nsp7, and nsp8 at a ratio of 1:5:1:1 provides the optimal conditions for subsequent inhibitor screening.

### 2.3. Functional Evaluation of a Dual-Reporter System for RdRp Activity Screening

Following optimization of the cell-based dual-reporter system, its performance was validated by screening fifteen alkaloid compounds with ribavirin included as positive treatment controls. Several compounds in this class have been reported to exhibit broad-spectrum antiviral activity against RNA viruses, including inhibition of RdRp [28,29]. Prior to screening, the cytotoxicity of all compounds was evaluated in both BHK-21 and CRFK cells to determine their half-maximal cytotoxic concentration (CC_50_) and to establish non-cytotoxic concentrations for subsequent assays (Table 1). The CC_50_ values varied between the two cell lines, indicating cell line-dependent differences in compound cytotoxicity.

**Figure 3:**
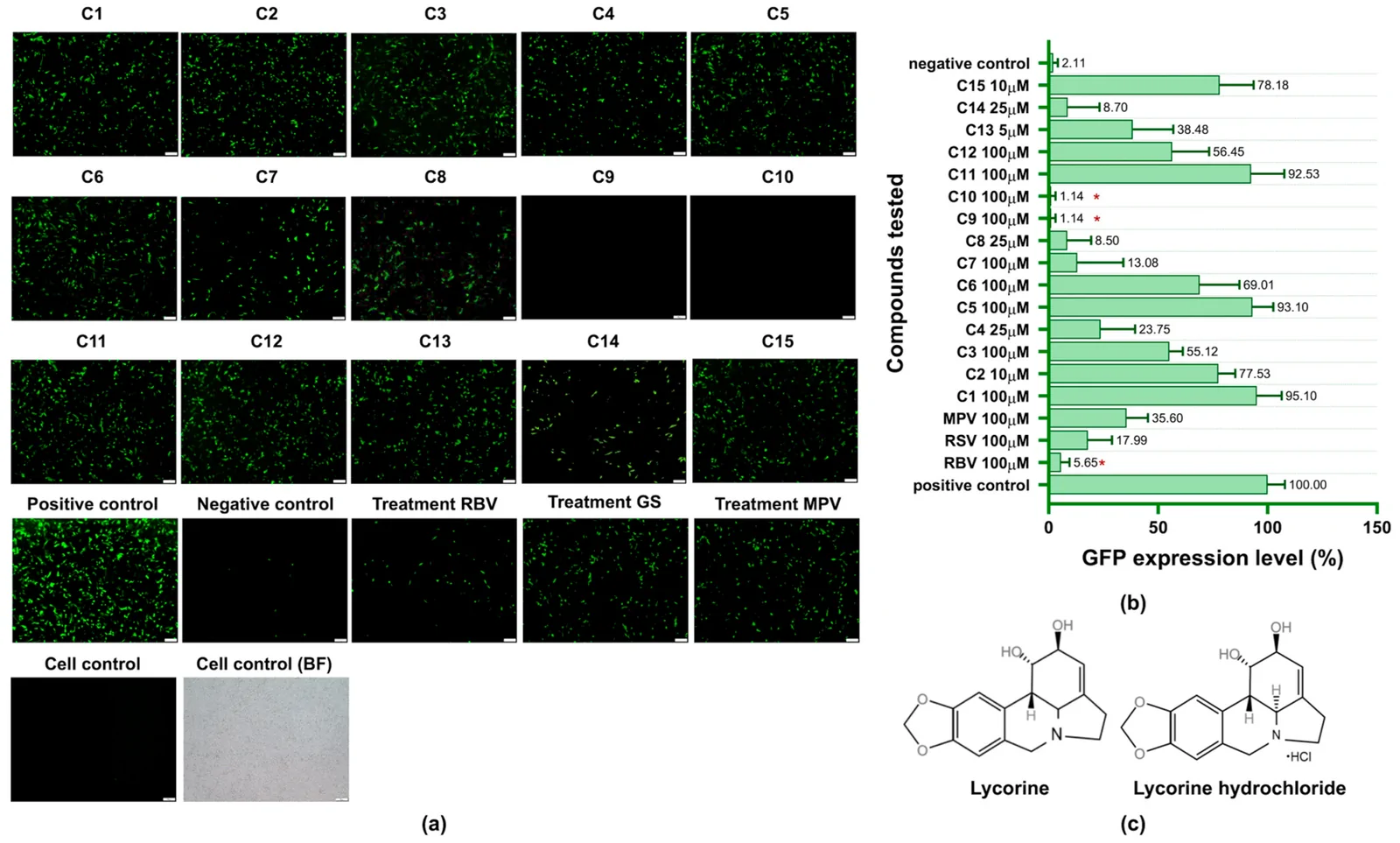
Screening of fifteen compounds for FIPV RdRp inhibition using the cell-based dual-reporter assay. (**a**) Compounds C1–C15 were screened in BHK-21 cells co-transfected with the dual reporter and FIPV RdRp-complex plasmids; GFP signals reflecting FIPV RdRp-dependent reporter activity were assessed at 24 hpt. Scale bars, 100 µm. (**b**) GFP signals quantified by image analysis (CellProfiler v4.2.8) and expressed as the percentage of GFP-positive cells; of the fifteen compounds, only two significantly reduced GFP expression compared with the positive control. The red asterisk (*) indicates a statistically significant difference at *p* < 0.05. (**c**) Chemical structures of the two active alkaloids identified in the primary screening.

To evaluate inhibitory activity against FIPV RdRp-dependent reporter activity, BHK-21 cells co-expressing the dual-reporter construct and the FIPV RdRp complex were treated with each test compound at concentrations selected based on the corresponding cytotoxicity results in BHK-21 cells. GFP expression was then measured to determine the inhibitory effects of the compounds on RdRp activity. The primary screening identified only two of the fifteen compounds—lycorine (C9) and lycorine hydrochloride (C10)—that significantly reduced GFP expression compared with the positive control (0.01% DMSO) (*p* < 0.05) (Figure 3). Ribavirin, GS-441524, and molnupiravir were included as reference RdRp inhibitors. Although, all three reference compounds reduced GFP expression to below 50% of the control level at certain concentrations, only ribavirin produced a statistically significant reduction in GFP expression under the screening conditions (Figure 3b).

Because RFP fluorescence served as an internal control for transfection efficiency, the effects of the compounds on both GFP and RFP signals were evaluated. Treatment with lycorine and lycorine hydrochloride at 100 µM markedly reduced RFP fluorescence (Figure S3a), indicating that this concentration could interfere with transfection efficiency or cellular condition and potentially confound the interpretation of GFP-based reporter activity. However, the GFP/RFP ratio remained reduced, supporting a genuine decrease in reporter activity relative to the internal RFP control (Figure S3b,c). Accordingly, lower non-interfering concentrations were selected for the further characterization of lycorine and lycorine hydrochloride using the cell-based dual-reporter assay.

### 2.4. Evaluation of the Dual-Reporter System in a Dose-Response Analysis

To further evaluate the performance of the dual-reporter system, the two hit compounds, lycorine and lycorine hydrochloride, were subjected to dose-response analysis. Ribavirin, GS-441524, and molnupiravir—three clinically approved antiviral agents with reported activity against RNA viruses—were included as reference compounds. BHK-21 cells co-expressing the dual-reporter construct and the FIPV RdRp complex were treated with serial dilutions of each compound, and RdRp-dependent reporter activity was assessed by measuring GFP fluorescence intensity.

**Figure 4:**
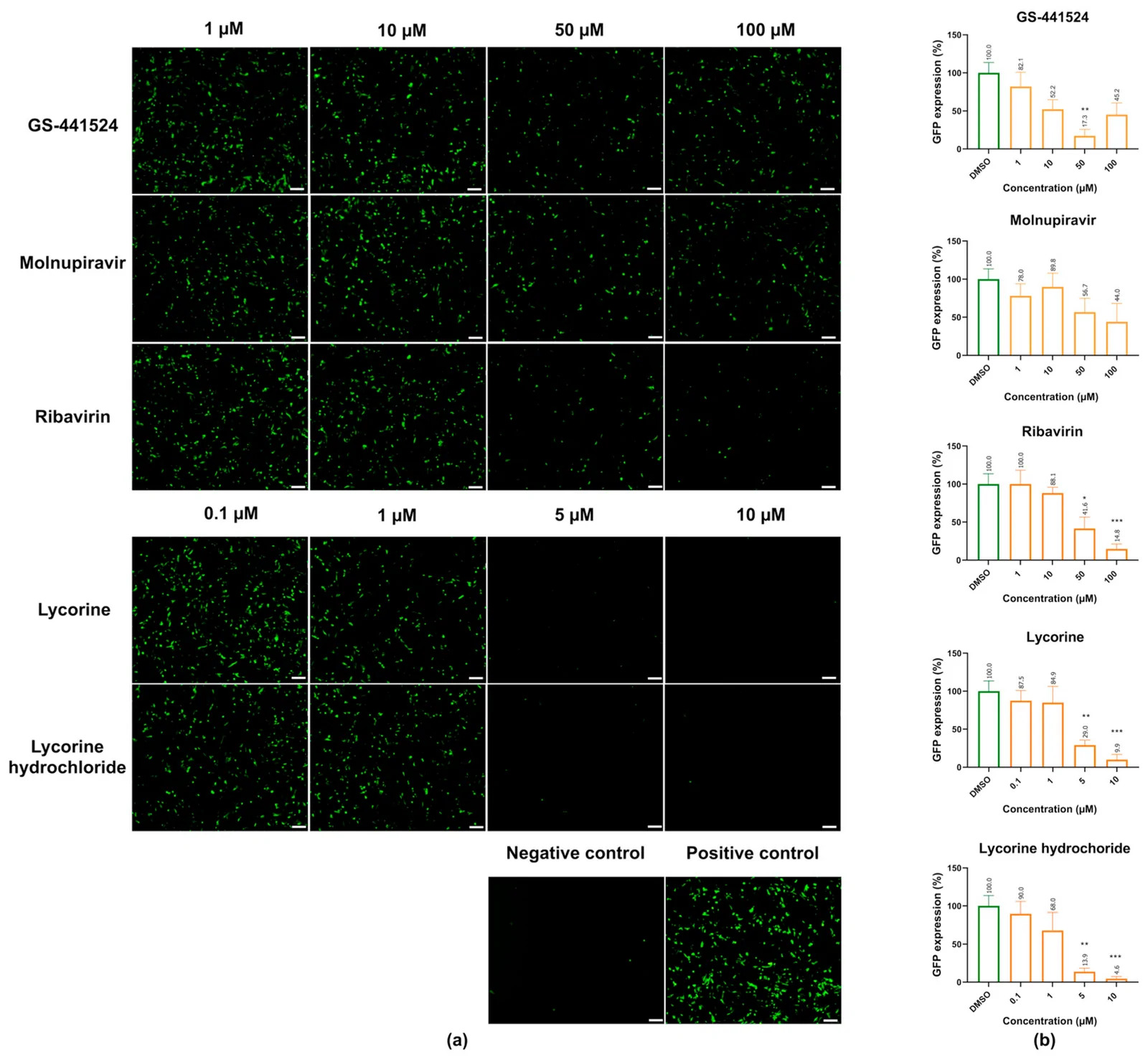
Inhibitory effects of selected compounds on FIPV RdRp activity measured with the cell-based dual-reporter assay. (**a**) Fluorescence images of GFP expression, reflecting FIPV RdRp-dependent reporter activity. Ribavirin, GS-441524, and molnupiravir served as reference RdRp inhibitors, while lycorine and lycorine hydrochloride were evaluated as candidate alkaloids. Cells co-transfected with the dual-reporter and FIPV RdRp-complex plasmids and treated with 0.01% DMSO served as the positive control, whereas cells transfected with the dual-reporter plasmid alone served as the negative control. Scale bars, 100 µm. (**b**) Concentration-dependent reduction in GFP-positive cells, expressed as the percentage relative to the positive control. Data are presented as mean ± SD. Statistical significance was defined as * *p* < 0.01, ** *p* < 0.005, and *** *p* < 0.001.

Three of the five tested compounds—ribavirin, lycorine, and lycorine hydrochloride—exhibited concentration-dependent inhibition of FIPV RdRp-dependent reporter activity. In contrast, GS-441524 and molnupiravir did not show a clear concentration-dependent response over the tested range; their IC_50_ values (Table 2) were therefore estimated and require further verification. GFP expression was normalized to the 0.01% DMSO vehicle control, defined as 100% reporter activity (Figure 4). Both lycorine and lycorine hydrochloride reduced GFP expression in a dose-dependent manner, with inhibitory activities comparable to that of ribavirin. Dose-response analysis yielded IC_50_ values of 2.83 ± 0.45 µM for lycorine, 1.58 ± 0.20 µM for lycorine hydrochloride, 38.25 ± 1.58 µM for ribavirin, whereas the estimated IC_50_ values for GS-441524 and molnupiravir were 13.40 ± 1.13 µM and 82.60 ± 1.92 µM, respectively. The RFP signal and GFP/RFP ratio were also evaluated for all tested compounds (Figure S4). Most compounds showed no significant effect on the RFP signal expression in the BHK-21 cells, except for lycorine hydrochloride, which reduced RFP fluorescence at highest concentration (10 µM). Overall, these results indicated that the cell-based dual-reporter assay can reliably detect concentration-dependent inhibition of FIPV RdRp activity and differentiated compounds according to their inhibitory potencies.

### 2.5. Validation of Dual-Reporter System and Assessment of Assay Quality

**Figure 5:**
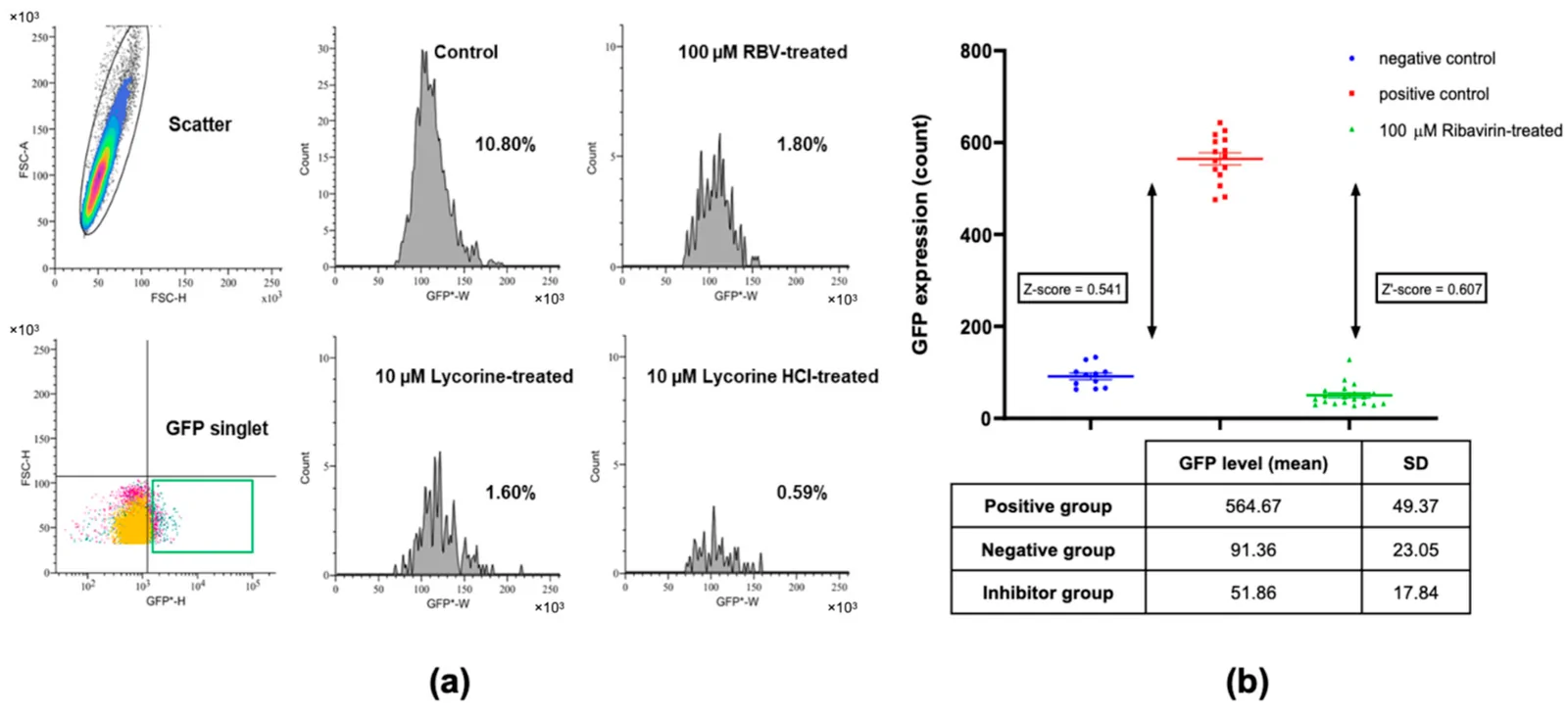
Validation of the dual-reporter assay in BHK-21 cells by flow cytometry and assay performance analysis. (**a**) Flow cytometry gating strategy used to quantify GFP-positive cells. Forward scatter (FSC) area and height were applied sequentially to exclude aggregates and identify single cells, and GFP fluorescence was analyzed within the singlet population. The percentage of GFP-positive cells was determined for transfected control and compound-treated cells within the GFP singlet gate. (**b**) Assay evaluation for potential HTS using Z- and Z′-factor analysis. Controls comprised cells transfected with the dual-reporter plasmid alone (negative control), cells co-transfected with the dual-reporter and FIPV RdRp-complex plasmids (positive control), and cells treated with 100 µM ribavirin (control drug). At 24 hpt, GFP expression was quantified by fluorescence image analysis (CellProfiler v4.2.8).

To validate the dual-reporter platform at the single-cell level, transfected BHK-21 cells were analyzed by flow cytometry following treatment with selected RdRp inhibitors. Cell populations were first gated to exclude debris and non-viable cells, then analyzed based on GFP fluorescence intensities (Figure 5a). Cells co-expressing the dual-reporter construct and the RdRp complex exhibited 10.80% GFP-positive cells. Treatment with 100 µM ribavirin reduced the GFP-positive population to 1.80%, a 6-fold decrease relative to the vehicle control. Similarly, 10 µM lycorine and 10 µM lycorine hydrochloride reduced the GFP-positive population to 1.60% and 0.59%, corresponding to 6.75-fold and 18.30-fold reductions, respectively. Ribavirin, lycorine and lycorine hydrochloride slightly reduced RFP fluorescence; nevertheless, the preferential reduction in GFP signal, together with the relatively preserved RFP signal, supports the ability of the dual-reporter system to distinguish RdRp-dependent reporter inhibition from nonspecific effects associated with excessive compound concentrations. These findings further support the utility of the dual-reporter system for monitoring FIPV RdRp activity.

We next examined whether the assay is suitable for high-throughput screening (HTS) by evaluating its robustness and reproducibility using the Z-factor and Z′-factor, as applied in previously reported cell-based RdRp reporter assays [18,19,22] and based on the framework of Zhang et al. [30]. The Z-factor, which reflects the discriminatory ability of the assay, was determined from the negative control (dual-reporter plasmid alone) and the positive control (dual-reporter plasmid co-transfected with the FIPV nsp12/nsp7/nsp8 complex). The Z′-factor, which reflects the applicability of ribavirin as a reference inhibitor, was determined from the ribavirin-treated group (100 µM) and the positive control. Under the optimized assay conditions, the dual-reporter platform achieved a Z-factor of 0.541 and a Z′-factor of 0.607 (Figure 5b). According to the criteria established by Zhang et al. [30], assays with Z-factor or Z′-factor values between 0.5 and 1.0 are considered excellent for HTS. These values therefore indicate that the dual-reporter assay is potentially suitable for HTS and could serve as a platform for the identification and quantitative evaluation of FIPV RdRp inhibitors.

### 2.6. Antiviral Efficacy of Candidate RdRp Inhibitors Against FIPV

**Figure 6:**
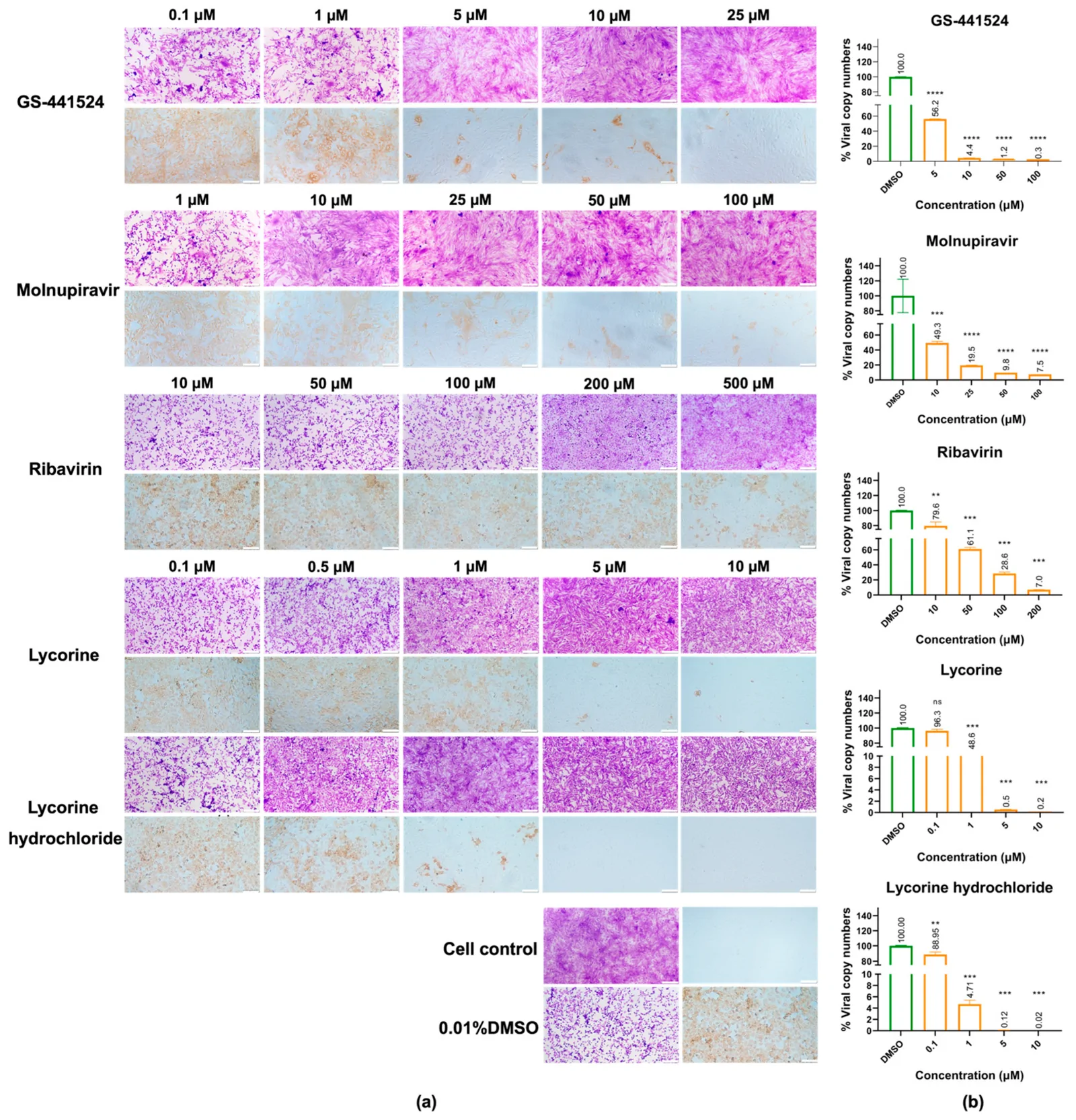
Antiviral activity of selected alkaloids against FIPV in infected CRFK cells. (**a**) Crystal violet staining showing FIPV-induced cytopathic effects (CPE), and IPMA detecting FIPV antigens in the cytoplasm of infected cells. Lycorine and lycorine hydrochloride reduced the number of FIPV-positive cells in a dose-dependent manner. Ribavirin, GS-441524 and molnupiravir were included as reference antiviral controls; non-infected cells and infected cells treated with 0.01% DMSO served as the cell and virus controls, respectively. Scale bars, 100 µm. (**b**) Dose-dependent inhibition of FIPV replication quantified by RT-qPCR. Data are mean ± SD. ** *p* < 0.01, *** *p* < 0.001, and **** *p* < 0.0001; ns, not significant.

To validate the antiviral activity of the candidate RdRp inhibitors identified by the dual-reporter assay, the two selected compounds, lycorine and lycorine hydrochloride, were further evaluated against infectious FIPV in CRFK cells. Ribavirin, GS-441524, and molnupiravir were included as reference antiviral compounds. Antiviral efficacy was initially assessed by IPMA to determine the half-maximal effective concentration (EC_50_) (Figure 6a). Both lycorine and lycorine hydrochloride inhibited FIPV infection in a concentration-dependent manner, with EC_50_ values of 0.81 ± 0.09 µM and 0.40 ± 0.04 µM, and high selectivity indices of 40.5 and 51.0, respectively (SI > 10). Under the same experimental conditions, ribavirin, GS-441524, and molnupiravir exhibited an EC_50_ of 105.90 ± 2.03 µM, 2.61 ± 0.42 µM and 12.99 ± 1.11 µM, respectively (Table 2). The antiviral activities of the selected compounds were further confirmed by quantitative RT-PCR analysis of viral RNA and determine the IC_50_ (Table 2). Consistent with the IPMA results, treatment with lycorine and lycorine hydrochloride produced concentration-dependent reductions in viral RNA levels. At concentrations of 5 µM, lycorine and lycorine hydrochloride reduced viral RNA copy numbers by 94.7% and 99.9%, respectively, relative to the untreated control (Figure 6b). These findings demonstrate that the two compounds screened by the dual-reporter platform effectively inhibit FIPV replication in cell culture. Moreover, the strong agreement between the dual-reporter assay and antiviral efficacy against whole virus supports the utility of this platform as a reliable and predictive tool for identifying FIPV RdRp inhibitors with functional antiviral activity.

## 3. Discussion

The treatment of FIP has been significantly advanced by GS-441524 and its prodrug remdesivir, both of which act as nucleoside analogs that suppress viral replication through RdRp and have demonstrated high therapeutic efficacy [3,15,16,31,32,33]. More recently, molnupiravir, another RdRp-targeting nucleoside analog, has shown comparable efficacy and safety in cats with FIP [15,16,31,32,33]. Despite these advances, access to GS-441524 and remdesivir remains limited in some regions, and their use have been associated with adverse injection-site reactions [3,16]. Furthermore, the risk of emerging drug-resistant variants [34] and the inherent limitations of current antivirals reinforce the need for continued identification of new RdRp inhibitors. Given that RdRp is indispensable for coronavirus replication [18,19,22,27], developing efficient and reliable screening platforms represents a direct strategy for advancing FIPV antiviral discovery.

In the present study, we developed and validated a cell-based dual-reporter system for live-cell monitoring FIPV RdRp activity. Unlike conventional luciferase-based assays that require substrate addition or cell lysis, this fluorescence-based platform enables real-time, quantitative detection of RdRp-dependent reporter activity in intact cells, with added compatibility for live-cell imaging and high-throughput screening applications. These features position the dual-reporter system as a practical and accessible platform for the identification and characterization of candidate RdRp inhibitors.

The dual-reporter system comprises the RFP gene in the forward orientation and the GFP gene in reverse orientation. Transcription is driven by the CMV promoter, and the transcription is subsequently processed by ribozyme-mediated self-cleavage to generate an RNA containing the FIPV-3′UTR-GFP-5′UTR in the negative-sense orientation. This design prevented GFP expression from plasmid-derived transcription, as the reverse orientation of the GFP gene does not permit direct translation by the host cellular machinery [18,19]. In contrast, expression of the RFP reporter serves as a transfection control. Importantly, the signal from the reverse-oriented GFP arises exclusively from RNA synthesized de novo by RdRp in -trans, enabling a specific and reliable assessment of RdRp activity [18]. The functional assay efficiency was further validated and optimized by measuring of GFP signals in the presence and absence of RdRp complex in BHK-21 cells. The use of the complete RdRp complex (nsp12, nsp7, and nsp8) resulted in higher GFP reporter activity than nsp12 alone, supporting the inclusion of all three subunits in our system. Although the magnitude of this enhancement was relatively modest, it is consistent with previous observations that the contribution of nsp7 and nsp8 to coronavirus RdRp-dependent reporter activity can vary depending on the experimental system employed [18,19]. Nonetheless, the improved reporter signal observed with the complete complex aligns with established structural and biochemical evidence demonstrating that nsp7 and nsp8 act as essential cofactors that stabilize nsp12 and promote efficient viral RNA synthesis [8,24,35,36], further supporting the rationale for using the full RdRp complex in our cell-based assay.

The enhanced reporter activity likely reflects the increased polymerase efficiency conferred by the formation of a complete functional RdRp complex, an effect particularly relevant in the context of FCoV, which exhibits several structural and biochemical features distinct from other coronaviruses. In contrast to the hexadecameric nsp7–nsp8 complex reported for SARS-CoV, FCoV nsp7 and nsp8 assemble into a 2:1 heterotrimeric complex [9]. Furthermore, FCoV nsp8 possesses noncanonical RNA polymerase activity capable of de novo (primer-independent) RNA synthesis. This activity is substantially enhanced by nsp7, allowing synthesis of RNA products that extend to the full template length [9]. These findings suggest that nsp7 and nsp8 contribute not only to polymerase processivity but may also directly influence RNA synthesis during FCoV replication. The precise roles of nsp7 and nsp8 may, however, vary across alphacoronaviruses depending on the experimental system employed [37]. Our cell-based results nonetheless demonstrate that co-expression of nsp7 and nsp8 substantially enhances FIPV RdRp-dependent reporter activity, supporting their indispensable roles in the functional polymerase complex during intracellular viral RNA synthesis. Based on these findings, we selected co-expression of nsp12, nsp7, and nsp8 as the optimized configuration for all subsequent experiments. Incorporating the complete RdRp complex not only increased assay sensitivity but also provided a more biologically relevant platform for evaluating inhibitors targeting the FIPV replication machinery.

To further validate dual-reporter system, three well-characterized nucleoside analogs targeting viral RdRp—ribavirin, GS-441524 and molnupiravir—were evaluated. Ribavirin is a synthetic guanosine analog with broad-spectrum antiviral activity against RNA viruses [38], has been reported to exert both direct and indirect inhibitory effects on FCoV replication [39]. Its activity in BHK-21 cells has been demonstrated against multiple viruses, including FMDV [13,40] and VSV [41], indicating that these cells efficiently metabolize ribavirin into its active phosphorylated forms. GS-441524 and molnupiravir are well-established therapeutic options for FIP [15,16,31,32,33,42]; GS-441524 inhibits coronavirus RdRp-mediated RNA synthesis, whereas molnupiravir induces lethal mutagenesis during viral RNA replication [15,42]. All three were included as reference inhibitors in the dual-reporter assay, alongside lycorine and lycorine hydrochloride, which were identified as candidate inhibitors from the primary screening.

Although GS-441524 and molnupiravir reduced FIPV RdRp-dependent reporter activity by approximately 55% at the highest concentration tested, neither exhibited a clear concentration-dependent response. This may reflect limited intracellular activation, as their antiviral effects require phosphorylation to generate active nucleoside triphosphate metabolites—a rate-limiting step that varies across cell types [43,44,45]. In contrast, ribavirin produced a consistent concentration-dependent response, supporting its use as a reference inhibitor under these assay conditions. Lycorine and lycorine hydrochloride exhibited substantially lower IC_50_ values than ribavirin, indicating greater potency in suppressing FIPV RdRp-dependent reporter activity.

Lycorine is a bioactive pyrrolidine alkaloid isolated from the bulbs of *Lycoris radiata* [28,29,46] has been explored for its antitumor and antiviral properties in both its free base and hydrochloride salt forms [47]. Lycorine has demonstrated broad-spectrum antiviral activity in vitro against multiple viruses, including poliovirus, SARS-CoV, herpes simplex virus type 1, bunyaviruses, Zika virus, and SARS-CoV-2 [14,29,48,49,50,51,52]. Mechanistically, lycorine inhibits RdRp activity in a dose-dependent manner, likely through binding to the finger domain of the polymerase with EC_50_ values of 0.39 and 0.22 µM in Vero and A549, Huh7 cells, respectively [14]. A cell-based reporter assay for MERS-CoV further demonstrated that lycorine acts as a non-nucleoside RdRp inhibitor without substantially interfering with host cellular transcriptional or translational machinery at concentrations up to 3.6 µM [53]. In the present study, both lycorine and lycorine hydrochloride exhibited comparable concentration-dependent inhibition of FIPV RdRp-GFP activity, suggesting that the lycorine scaffold may contribute to the observed antiviral activity regardless of the salt form. However, lycorine hydrochloride showed lower reporter IC_50_ and antiviral EC_50_ values and a somewhat higher selectivity index than lycorine free base, indicating differences in their antiviral activity and selectivity.

The characterization of these alkaloid compounds with RdRp inhibitory activity further validated the utility of the dual-reporter system as a screening platform. Both compounds reduced reporter activity in a dose-dependent manner, and their IC_50_ values were consistent with the EC_50_ values obtained from antiviral assays against FIPV, supporting the ability of the assay to predict antiviral activity through RdRp inhibition measurement. Despite these promising results, several limitations of the system should be considered. First, although the dual-reporter design was intended to restrict GFP production to FIPV RdRp-dependent transcription, a measurable baseline GFP signal was observed in cells transfected with the reporter plasmid alone. Similar background signals have been reported in previous coronavirus RdRp reporter systems [18,19] and may arise from low-level host RNA polymerase activity facilitated by cryptic promoter-like elements within the viral UTRs, making some degree of basal expression likely unavoidable. Importantly, the background did not compromise assay performance. The assay showed good reliability and reproducibility (Z- and Z′-factor > 0.5), supporting its utility as a practical cell-based tool for identifying inhibitors of FIPV RdRp-dependent reporter activity. Second, as the assay was validated in BHK-21 cells, compound performance may vary in other cell types owing to differences in cellular uptake, metabolism, or cytotoxicity [45,53], and promising candidates should be further validated in cell-based whole-virus infection assays and in vivo models. A further limitation applies to compounds that require intracellular metabolic activation. Consistent with this, GS-441524 and molnupiravir showed antiviral activity in the whole-virus assay but did not produce a conventional concentration-dependent response in the reporter system, indicating that their activity may not fully captured by this platform. Results for metabolically dependent compounds–whether positive or negative–should therefore be interpreted with caution and confirmed using complementary assays or cell systems with appropriate metabolic capacity.

In conclusion, the cell-based dual-reporter assay provides an effective approach for assessing FIPV RdRp-dependent reporter activity in BHK-21 cells and screening candidate antiviral compounds. Given the high conservation of RdRps among RNA viruses, this platform provides a practical foundation for FIPV antiviral screening and may serve as a starting point for developing related cell-based screening systems for other RNA viruses.

## 4. Materials and Methods

### 4.1. Cells, Viruses, and Compounds

Baby hamster kidney cells (BHK−21; ATCC^®^, Manassas, VA, USA) were used for dual-reporter assays, cytotoxicity assay and RdRp complex expression. Crandell–Rees feline kidney cells (CRFK; ATCC^®^, Manassas, VA, USA) were used for virus propagation, cytotoxicity assay and all experiments involved viral infection. Cells were cultured in Minimum Essential Medium (MEM; Gibco™; Thermo Fisher, Waltham, MA, USA) supplemented with 10% fetal bovine serum (FBS; Gibco™; Thermo Fisher, Waltham, MA, USA), 2 mM L-glutamine, and 1% ampicillin at 37 °C in 5% CO_2_.

Feline infectious peritonitis virus (FIPV; WSU 79-1146, ATCC^®^) was propagated in CRFK cells as previously described [54,55,56]. Viral titers were determined by Reed and Muench’s method [57] and expressed as the 50% tissue culture infectious dose (TCID_50_/mL). The virus stock with a titer of 1 × 10^6.5^ TCID_50_/mL was stored at −80 °C for subsequent experiments.

The fifteen alkaloid compounds (Table 1) were purchased from ChemFaces (Wuhan, China). Ribavirin, GS-441524 and molnupiravir (Sigma-Aldrich^®^, St. Louis, MO, USA) was used as RdRp inhibitor controls. All compounds were prepared as 10 mM stock solutions in 100% dimethyl sulfoxide (DMSO) (Sigma-Aldrich^®^, St. Louis, MO, USA) and stored at −20 °C for the following in vitro cell-based experiments.

### 4.2. Establishment of a Dual-Reporter Plasmid

To generate the dual-reporter plasmid, we adapted a previously described bicistronic RdRp reporter construct [19,22]. A gene construct was synthesized, consisting of the mCherry (GenBank accession no. AY678264), a hepatitis delta virus (HDV) ribozyme, followed by a reverse orientation cassette of the 3′-UTR of FIPV (GenBank accession no. DQ010921), enhanced green fluorescent protein (eGFP; GenBank accession no. CVU55762), the 5′-untranslated region (5′-UTR) of FIPV (GenBank accession no. DQ010921), and a second HDV ribozyme via GenScript (Piscataway, NJ, USA). The synthesized fragment was cloned into the pcDNA3.1 (+) vector through subcloning using *Eco*RI and *Xho*I which located upstream of the mCherry gene and downstream of the second HDV ribozyme sequence, to generate the dual-reporter plasmid.

### 4.3. Construction of FIPV RdRp Complex-Derived Plasmids

FIPV was extracted using TRIzol reagent (Invitrogen™, Carlsbad, CA, USA) and viral RNA was reverse transcribed to cDNA using RevertAid reverse transcriptase (Thermo Fisher, Waltham, MA, USA) which served as the template for PCR amplification. The primers for construction of N-terminal (N-term) His-tagged FIPV nsp12 gene (RdRp), His-tagged nsp7 gene, and His-tagged nsp8 gene (GenBank accession no. DQ010921) were designed (Table S1). The amplicon sizes for the nsp12, nsp7, and nsp8 genes were 2783, 249, and 585 base pairs (bp), respectively. Each fragment was then subsequently cloned into the *Xma*I and *Xho*I sites of the pCAGGS vector (Invitrogen™, Carlsbad, CA, USA) to generate, pHis-nsp12, pHis-nsp7 and pHis-nsp8, respectively.

### 4.4. Detection of FIPV RdRp Complex Protein Expression by Immunoperoxidase Monolayer Assay (IPMA)

To confirm RdRp complex proteins expression in cell culture, IPMA was performed as described previously [58]. Briefly, BHK-21 cells were seeded in 96-well plates and cultured overnight. Cells were subsequently transfected in triplicate with pHis-nsp12, pHis-nsp7 or pHis-nsp8 and the empty vector (pCAGGS) served as a negative control. At 48 hpt, cells were fixed with cold methanol for 30 min at room temperature and washed three times with 1× PBST (phosphate-buffered saline containing 0.1% Tween 20; Sigma-Aldrich^®^, St. Louis, MO, USA). Cells were then blocked with BlockPRO™ 1 Min Protein-Free Blocking Buffer (Taipei, Taiwan) for 1 min, followed by three washes with 1× PBST. The fixed cells were incubated with anti-histidine tagged antibody (1:500, Sigma-Aldrich^®^, St. Louis, MO, USA) at 37 °C for 1 h. After washing three times with 1× PBST, cells were incubated with HRP-conjugated goat anti-mouse IgG antibody (1:1000, Sigma-Aldrich^®^, St. Louis, MO, USA) at 37 °C for 1 h. Following three washes with 1× PBST, cells were incubated with DAB substrate (DAKO, Santa Clara, CA, USA). Dark-brown intracytoplasmic staining was observed under an inverted microscope (Olympus IX73, Tokyo, Japan).

### 4.5. Optimization of Cell-Based Dual-Reporter Assay

A dual-reporter system was conducted in BHK-21 cells. Cells at 2.0 × 10^4^ cells/well were seeded in 96-well plates and incubated overnight at 37 °C. Transfection was performed using Fugene^®^ HD (Promega, Madison, WI, USA) as the transfecting reagent to introduce the dual-reporter plasmid, either single transfection reporter plasmid or in plasmid combination various ratio with FIPV nsp12 (RdRp), nsp7 and nsp8 or co-transfection with various ratio of only nsp12. After 15-min incubation at room temperature, the transfection mixture was added into the cells and were then incubated at 37 °C with 5% CO_2_. The dual reporter activities were assessed at 24 h post-transfection (hpt), by detecting fluorescence signals using an inverted microscope (Olympus IX73, Japan) using blue and green excitation for GFP and RFP, respectively, and bright-field illumination for cell visualization. The levels of FIPV RdRp activity were quantified based on the numbers and intensity of GFP-positive cells, while RFP expression served as a transfection control. The relative activity of FIPV RdRp was determined by normalizing the level of GFP to that of RFP (GFP/RFP ratio). Fluorescence images were analyzed using CellProfiler (v4.2.8, Broad Institute, Cambridge, MA, USA) to quantify fluorescence cells, and background signal was subtracted from each image as previously described [13]. Statistical analyses were performed using ordinary one-way analysis of variance (ANOVA), followed by Tukey’s multiple-comparison test. Three independent biological replicates, each with triplicate technical replicates were analyzed for each group, and the mean of the technical replicates was used for statistical analysis.

### 4.6. Cytotoxicity Assay

The BHK-21 cells or CRFK cells were seeded in 96-well plate at a density of 2.0 × 10^4^ cells/well overnight and were subsequently treated with serial dilutions of each compound (0.1, 1, 10, 25, 50, 100, and 200 µM in a final volume 100 µL per well) [13,54,55,56]. Cells treated with 0.01% DMSO alone served as the vehicle control and were defined as representing 100% cell viability. Cell viability was assessed after 24 h using the CCK-8 assay (10 µL per well), incubated at 37 °C for 2 h according to manufacturer’s instruction. The OD at 450 nm was measured using a Synergy H1 Hybrid Multi-Mode Reader (BioTek Instruments, Winooski, VT, USA) and normalized to the DMSO control. The CC_50_ was defined as the compound concentration required to reduce cell viability by 50%. CC_50_ values were determined by nonlinear regression (log[inhibitor] vs. response) using GraphPad Prism (version 8.4.0; GraphPad Software, San Diego, CA, USA).

### 4.7. Screening of Alkaloid Compounds Using the Dual-Reporter System

A dual-reporter cells-based screening assay was performed to evaluate the inhibitory effects of fifteen alkaloid compounds on FIPV RdRp activity. BHK-21 cells at 2 × 10^4^ cells/well were seeded in 96-well plates and incubated overnight. Cells were transfected with a mixture containing dual-reporter plasmid together with FIPV nsp12, nsp7 and nsp8, followed by treatment with ribavirin and alkaloids at a single concentration selected based on cytotoxicity evaluation in BHK-21 cells. The concentration of each compound is shown in Figure 3b. Wells treated with 0.01% DMSO served as transfection-positive controls, while cells transfected with the dual-reporter plasmid alone (without FIPV RdRp) served as negative controls. Ribavirin, GS-441524, and molnupiravir were included as reference RdRp inhibitors. The final DMSO concentrations in the compound-treated wells ranged from 0.001% to 0.1% (*v*/*v*). Cells were incubated at 37 °C with 5% CO_2_, and fluorescence signals were observed at 24 hpt using an inverted microscope and analyzed the signals as mentioned above.

### 4.8. Evaluation of Dose-Dependent Screening System

A dual-reporter cells-based screening assay was further performed to evaluate the dose-dependent inhibitory effects on FIPV RdRp activity. BHK-21 cells at 2 × 10^4^ cells/well were seeded in 96-well plates and incubated overnight. Cells were transfected with a mixture containing dual-reporter plasmid together with FIPV nsp12, nsp7 and nsp8, followed by treatment with ribavirin, GS-441524, molnupiravir and alkaloids at various concentrations. Wells treated with 0.01% DMSO served as transfection-positive controls, while cells transfected with the dual-reporter plasmid alone (without FIPV RdRp) served as negative controls. Cells were incubated at 37 °C with 5% CO_2_, and fluorescence signals were observed at 24 hpt using an inverted microscope and analyzed the signals as previously described above. The IC_50_ was calculated as the concentration of a compound that reduced FIPV RdRp activity by 50% relative to the DMSO control. IC_50_ values were determined by nonlinear regression (log[inhibitor] vs. response—variable slope) using GraphPad Prism (version 8.4.0; GraphPad Software, San Diego, CA, USA), with the DMSO control and the single dual-reporter transfection signal set as the 100% and 0% references, respectively. Each group was tested in three independent biological replicates, each performed with three technical replicates. Statistical analyses were performed using ordinary one-way ANOVA followed by Dunnett’s multiple-comparison test.

### 4.9. Validation of Cell-Based Dual-Reporter System by Flow Cytometry Analysis and Z- and Z′-Factor Determination

To confirm the cell-based dual-reporter transfection efficiency, GFP expression levels were analyzed by flow cytometry using a BD FACSMelody™ Cell Sorter (BD Life Sciences, Franklin Lakes, NJ, USA) At 24 hpt, culture media and transfection complexes were removed, and BHK-21 cells were washed once with 1× PBS. Cells were then detached using 0.05% Trypsin-EDTA (Gibco™; Thermo Fisher, Waltham, MA, USA), centrifuged at 2000× rpm for 5 min, and resuspended in 1 mL of 1× PBS. Up to 10,000 events were acquired per sample. Flow cytometric data were analyzed using standard gating procedures [59]. Live single cells were initially identified by forward scatter area (FSC-A) versus height (FSC-H) gating to exclude debris and doublets. Sequential gating for GFP and mCherry (RFP) fluorescence was then applied to determine reporter expression in each population.

To validate a dual-reporter assay, BHK-21 cells at 2 × 10^4^ cells/well were seeded in 96-well plates and incubated overnight. For the inhibitor treated reporter assay, cells were transfected with a mixture containing dual-reporter plasmid together with FIPV nsp12, nsp7 and nsp8 followed by treatment with ribavirin at various concentrations. 0.01% DMSO served transfection-positive control. Cells transfected with the dual-reporter plasmid alone (single transfection) were included as negative control. Cells were incubated at 37 °C with 5% CO_2_ for 24 hpt, after which GFP fluorescence was visualized and quantified to determine the percentage of GFP-positive cells. Experimental groups were defined as followed: (i) negative group, dual-reporter plasmid alone treated with 0.01% DMSO; (ii) positive group, dual-reporter plasmid co-transfected with FIPV nsp12, nsp7, nsp8 and treated with 0.01% DMSO; and (iii) inhibitor group, dual-reporter plasmid co-transfected with FIPV nsp12, nsp7, nsp8 and treated with 100 µM ribavirin. To assess assay reliability and reproducibility, the Z-factor, Z′-factor [18,19,22,30], and signal-to-background ratio were calculated from 20 replicate wells per group (positive control, negative control, and inhibitor-treated) within the same plate, in three independent experiments. The Z-factor was calculated using the negative and positive control groups whereas Z′-factor was calculated using the inhibitor and positive control groups following these equations:

$$Z-\mathrm{factor}=1-\frac{3\left({\mathrm{SD}}_{\mathrm{negative}}+SD_{\mathrm{positive}}\right)}{{\mathrm{Mean}}_{\mathrm{negative}}-{\mathrm{Mean}}_{\mathrm{positive}}}$$

$$Z^{\prime }-\mathrm{factor}=1-\frac{3\left({\mathrm{SD}}_{\mathrm{inhibitor}}+SD_{\mathrm{positive}}\right)}{{\mathrm{Mean}}_{\mathrm{inhibitor}}-{\mathrm{Mean}}_{\mathrm{positive}}}$$

### 4.10. Antiviral Assay and Viral Detection by Using IPMA

CRFK cells were seeded in 96-well plates at 2.2 × 10^4^ cells/well and incubated overnight. Cells were infected with FIPV (50 TCID_50_/mL) for 2 h, after which the inoculum was removed, cells were washed with 1× rinse saline, and fresh complete medium containing the test compounds was added. Antiviral activity of compound-treated, FIPV-infected cells, together with virus-infected and mock-infected controls was evaluated using cytopathic effect (CPE) reduction with 0.5% crystal violet staining at 48 hpi [55,56] and were further evaluated by IPMA as previously described [54,55,56]. The cells were fixed with cold methanol for 30 min and washed three time with 1× PBST. After blocking, the cells were incubated with mouse anti-FIPV3-70 (1:500; Thermo Fisher, Waltham, MA, USA) at 37 °C for 1 h, followed by HRP-conjugated goat anti-mouse IgG (1:500; Thermo Fisher, Waltham, MA, USA) under the same conditions. FIPV antigens were visualized using DAB substrate. Images were captured and further analyzed to quantify positive signals. Infection levels were normalized relative to controls, with mock-infected and FIPV-infected cells defined as 0% and 100% infection, respectively. The EC_50_ was defined as the compound concentration required to reduce viral infection by 50%. The EC_50_ values were determined by nonlinear regression using (log[inhibitor] vs. response—variable slope) GraphPad Prism (version 8.4.0; GraphPad Software, San Diego, CA, USA). Differences were considered significant at *p* < 0.05. The selectivity index (SI) was calculated as the ratio of CC_50_ to EC_50_.

### 4.11. Quantification of FIPV Copy Number by RT-qPCR

The CRFK cells were seeded in 24-well plates at 2 × 10^5^ cells/well and incubated overnight at 37 °C. Cells were infected with FIPV (50 TCID_50_/mL) for 2 h, after which the inoculum was removed then treated with compounds at the various concentrations and incubated at 37 °C for an additional 22 h. Viral RNA levels in compound-treated, FIPV-infected cells were quantified by RT-PCR and compared with virus-infected and mock-infected controls. Total RNA was extracted using TRIzol reagent (Invitrogen™, Carlsbad, CA, USA) and purified using the Direct-zol RNA MiniPrep kit (Zymo Research Corporation, Tustin, CA, USA). cDNA was synthesized from 1 µg of total RNA using RevertAid reverse transcriptase and random hexamers for viral load quantification [55]. qPCR was performed using iTaq Universal SYBR Green Supermix (2×), on a C1000 Touch Thermal Cycler (Bio-Rad Laboratories, Hercules, CA, USA) with primers listed in Table S1. Viral copy numbers were determined from a standard curve generated using 10-fold serial dilutions of a plasmid containing the FIPV 3′UTR sequence [54,55,56]. The IC_50_ was defined as the compound concentration required to reduce viral copy number by 50% relative to the DMSO control, which was set as the 100% reference. IC_50_ values were determined by nonlinear regression (log[inhibitor] vs. response—variable slope) using GraphPad Prism (version 8.4.0; GraphPad Software, San Diego, CA, USA). Each group was tested in three independent biological replicates, each performed in triplicate. Statistical analyses were performed using ordinary one-way ANOVA followed by Dunnett’s multiple-comparison test. Differences were considered significant at *p* < 0.05.

## Figures and Tables

**Table 1:** Cell cytotoxicity values of fifteen alkaloid compounds and references anti-RdRp compounds in BHK-21 and CRFK cells.

Code	Compounds	Cytotoxicity Assay (CC_50_, µM) ^1^	Cytotoxicity Assay (CC_50_, µM) ^2^
C1	Catharanthine	>200	107.4 ± 1.01
C2	Chelerythrine	15.17 ± 4.53	27.65 ± 4.88
C3	Crotaline	>200	109.2 ± 1.41
C4	Cyclovirobuxine	34.82 ± 2.64	334 ± 1.37
C5	Galantamine	>200	105.5 ± 1.69
C6	Galantamine hydrobromide	>200	139.4 ± 1.45
C7	Indirubin	>200	138.35 ± 1.34
C8	Liensinine	68.38 ± 1.54	169.19 ± 7.84
C9	Lycorine	180.90 ± 2.26	32.79 ± 2.46
C10	Lycorine hydrochloride	191.20 ± 2.28	20.43 ± 1.31
C11	Picrinine	>200	112.3 ± 0.67
C12	Protopine	178.70 ± 1.28	>200
C13	Sanguinarine chloride	8.65 ± 4.76	9.09 ± 5.142
C14	Tryptanthrine	33.18 ± 4.26	7.25 ± 0.86
C15	Ungeremine	29.41 ± 2.82	113.4 ± 2.00
**Reference Compounds ^3^**			
RBV	Ribavirin	>200	>500
GS	GS-441524	>100	173.59 ± 2.74
MPV	Molnupiravir	>100	114.4 ± 2.06

^1^ Cell cytotoxicity assay was evaluated in BHK-21 cells. ^2^ Cell cytotoxicity assay was evaluated in CRFK cells. ^3^ Clinically approved antiviral agents against RNA viruses.

**Table 2:** Inhibition and effective values of RdRp inhibitor compounds with 95% confidential interval (CI).

Compounds	IC_50_ Value (µM) ^1^	IC_50_ Value (µM) ^2^	EC_50_ Value (µM) ^3^	SI ^4^
GS-441524	13.40 ± 1.13 ^5^ (11.56 to 14.08)	1.13 ± 0.05 (1.09 to 1.17)	2.61 ± 0.42 (2.44 to 2.80)	66.50
Molnupiravir	82.60 ± 1.92 ^5^ (81.31 to 89.98)	9.57 ± 0.98 (8.64 to 10.44)	12.99 ± 1.11 (12.21 to 13.77)	8.80
Ribavirin	38.25 ± 1.58 (37.44 to 39.20)	53.98 ± 1.73 (53.2 to 54.76)	105.90 ± 2.03 (105.12 to 106.68)	>4.72
Lycorine	2.83 ± 0.45 (1.33 to 5.11)	0.98 ± 0.01 (0.74 to 1.76)	0.81 ± 0.09 (0.63 to 1.03)	40.48
Lycorine hydrochloride	1.58 ± 0.20 (0.60 to 3.22)	0.26 ± 0.06 (0.25 to 1.04)	0.40 ± 0.04 (0.16 to 0.74)	51.01

^1^ IC_50_ values were evaluated by cell-based dual reporter assay analysis. ^2^ IC_50_ values were evaluated by viral copy number analysis using RT-qPCR. ^3^ EC_50_ values were evaluated by antiviral activity assay analysis using IPMA. ^4^ Selectivity Index were calculated from CC_50_ and EC_50_ which evaluated in CRFK cells; SI = CC_50_/EC_50_. ^5^ Estimated values; these compounds did not exhibit a conventional concentration-dependent response.

## Data Availability

The original contributions presented in this study are included in the article/Supplementary Materials. Further inquiries can be directed to the corresponding author.

## Supplementary Materials

The following supporting information can be downloaded at: https://www.mdpi.com/article/10.3390/ijms27198682/s1. Reference [60] is cited in the Supplementary Materials.

## Abbreviations

The following abbreviations are used in this manuscript:

BHK-21	baby hamster kidney cells
CC_50_	half-maximal cytotoxic concentration
CCK-8	cell counting kit-8
CMV	cytomegalovirus
CRFK	Crandell–Rees feline kidney cells
DMSO	dimethyl sulfoxide
EC_50_	half-maximal effective concentration
FBS	fetal bovine serum
FCoV	feline coronavirus
FECV	feline enteric coronaviruses
FIPV	feline infectious peritonitis virus
FMDV	foot-and-mouth disease virus
GFP	green fluorescent protein
HDV	hepatitis delta virus
hpt	hour post-transfection
HTS	high-throughput screening
IC_50_	half-maximal inhibition concentration
IPMA	immunoperoxidase monolayer assay
MEM	minimum essential medium
MERS-CoV	Middle East respiratory syndrome coronavirus
PBST	phosphate-buffered saline containing 0.1% tween 20
PEDV	porcine epidemic diarrhea virus
RdRp	RNA-dependent RNA polymerase
RTC	replication–transcription complex
SARS-CoV2	severe acute respiratory syndrome coronavirus 2
UTR	untranslated region
VSV	vesicular stomatitis virus
ZIKV	Zika virus
