# Supplementary material for: Development and Application of a Dual-Fluorescent Reporter Assay for Feline Infectious Peritonitis Virus RNA-Dependent RNA Polymerase Inhibitor Screening
Source: Int J Mol Sci. 2026 Sep 28;27(19):8682. doi: 10.3390/ijms27198682 (PMC13665368; doi:10.3390/ijms27198682)
Supplement: Supplementary file 1 [file ijms-27-08682-s001.zip › ijms-4565360-supplementary.pdf]

Supplement materials

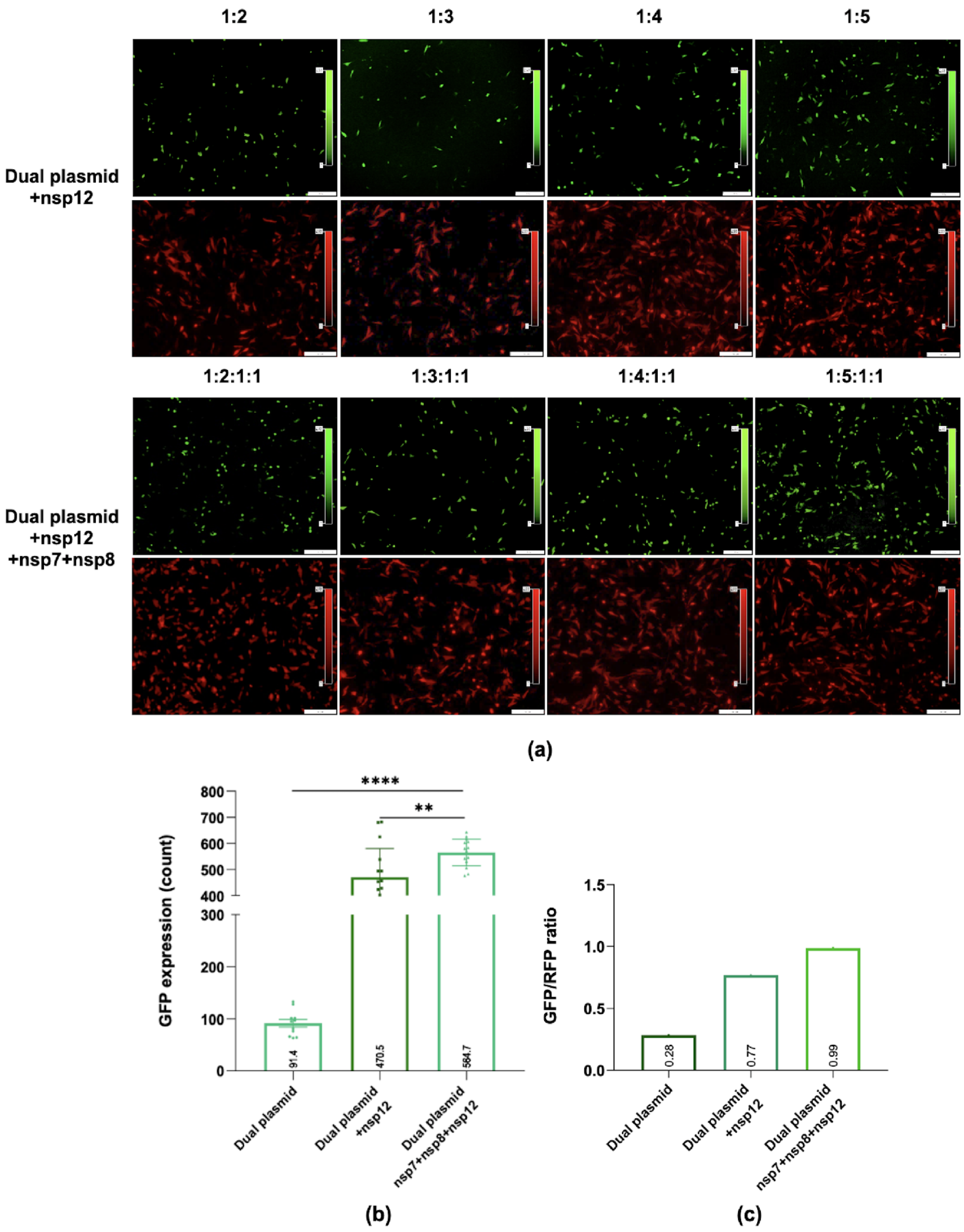

Figure S1. Optimization of the cell-based dual-reporter assay. (a) The dual-reporter plasmid was co-transfected with different amounts of FIPV nsp12 alone or in combination with nsp7 and nsp8 at the indicated ratios. GFP fluorescence, reflecting FIPV RdRp-dependent reporter activity, and RFP fluorescence, serving as an internal transfection control, were assessed at 24 h post-transfection (hpt) and quantified by image analysis using CellProfiler v4.2.8. Scale bars, 100 μm. (b) GFP fluorescence was quantified as GFP fluorescence counts. (c) RdRp activity was normalized as the GFP/RFP ratio. Data are presented as mean ± SD. Statistical significance is indicated as $^{**}p < 0.01$ and $^{****}p < 0.0001$.

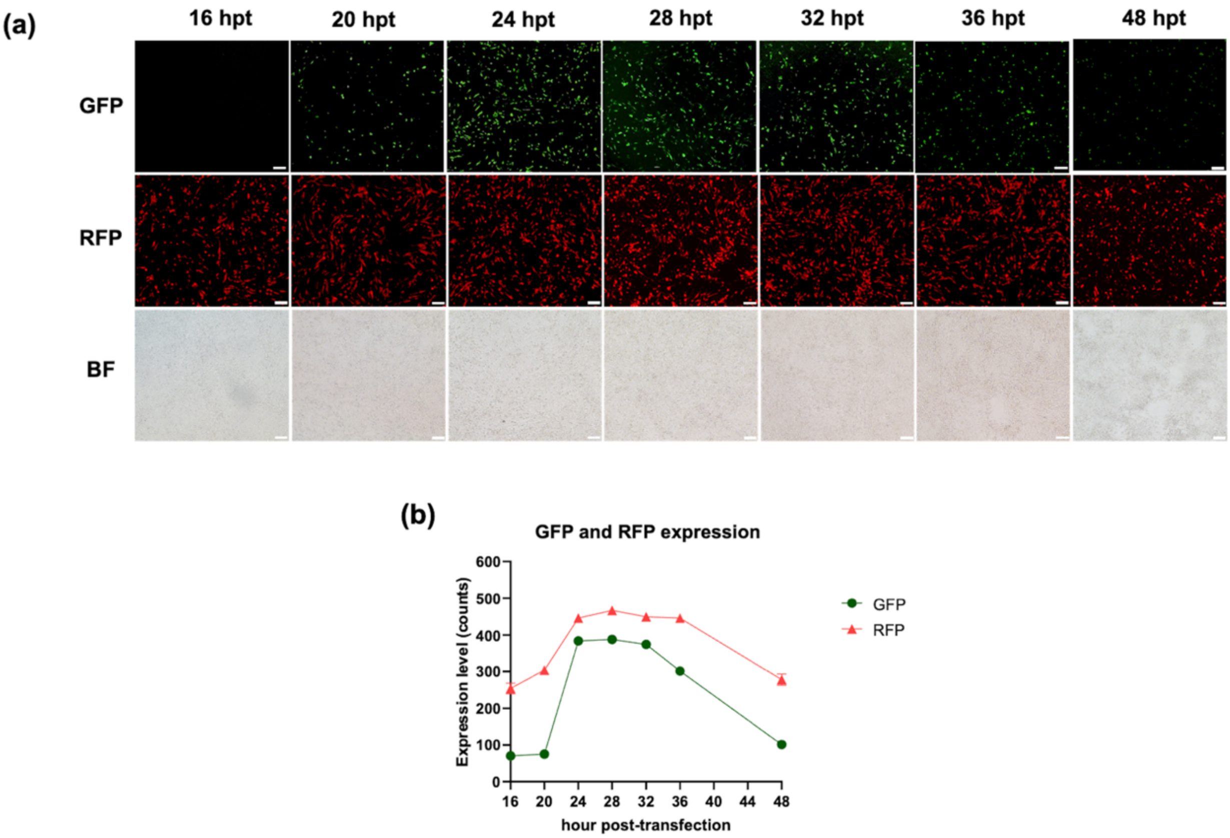

Figure S2. Time-course analysis of the cell-based dual-reporter assay. (a) Cells were co-transfected with the dual-reporter plasmid and the complete RdRp complex (nsp12, nsp7, and nsp8), and fluorescence was monitored at the indicated hours post-transfection (hpt). Scale bar, 100 μm. (b) GFP and RFP fluorescence intensities quantified by image analysis using CellProfiler v4.2.8 and reported as fluorescence counts. Data are presented as mean ± SD.

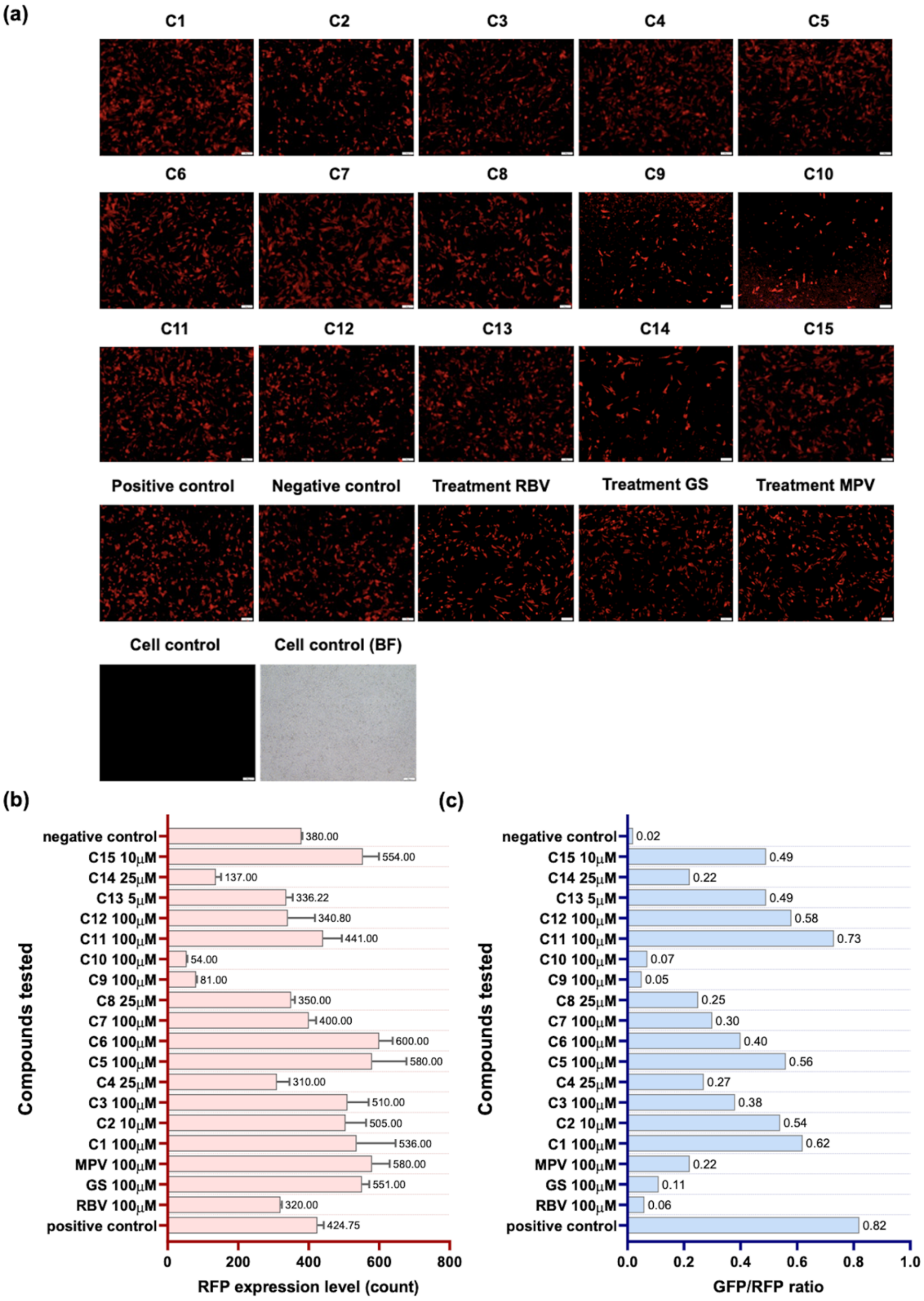

Figure S3. Effects of fifteen compounds on the internal RFP control in the cell-based dual-reporter assay. (a) Fifteen compounds (C1–C15) and reference RdRp inhibitors including—ribavirin (RBV), GS-441524 (GS) and molnupiravir (MPV)—were evaluated in BHK-21 cells co-transfected with the dual plasmid and FIPV RdRp-complex plasmids. RFP fluorescence was assessed at 24 h post-transfection (hpt). Scale bars, 100 μm. (b) RFP fluorescence counts and (c) GFP/RFP ratios were quantified to assess compound effects on the internal transfection control and RdRp-dependent reporter activity, respectively. Data are presented as mean ± SD.

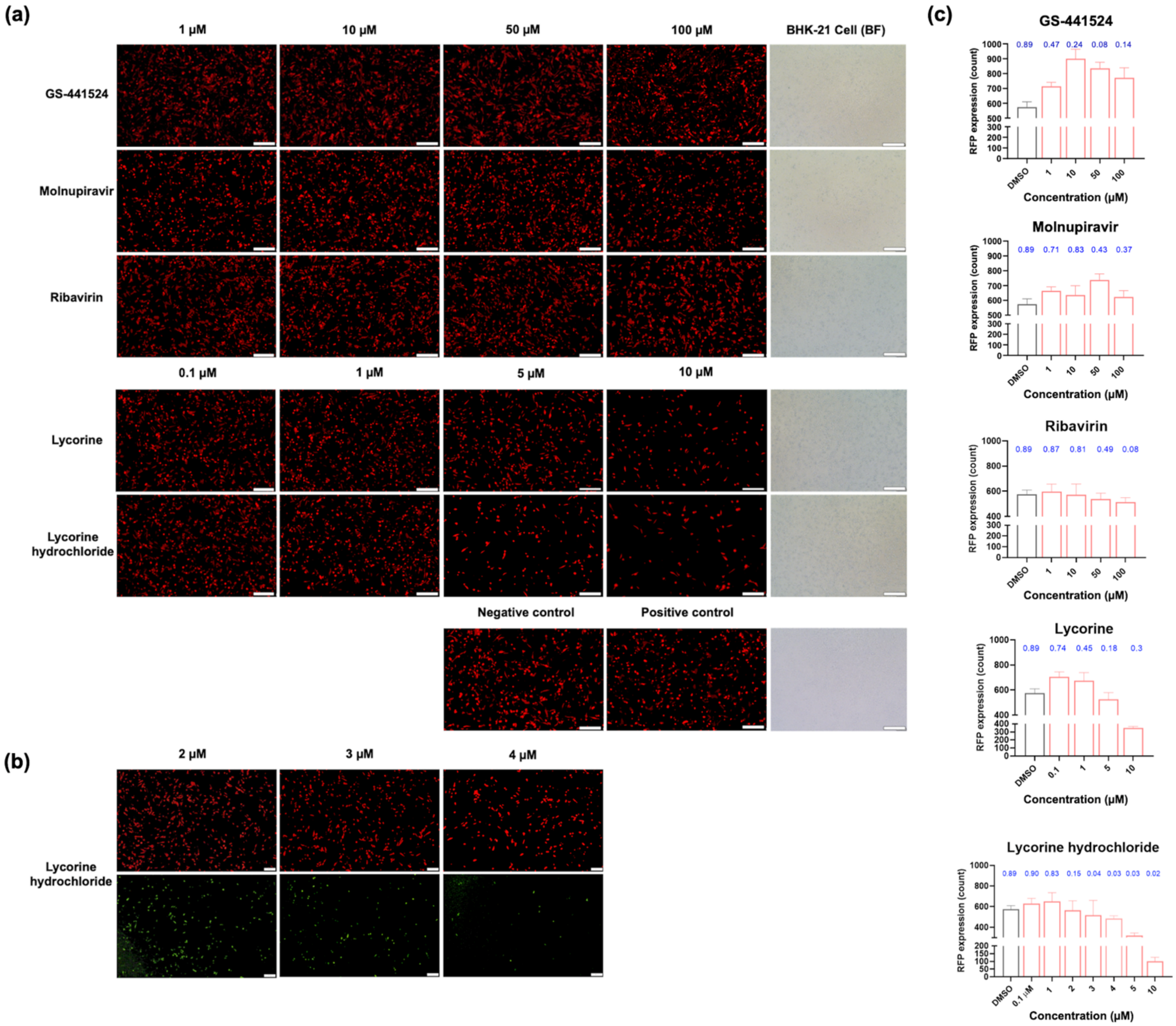

Figure S4. Effects of selected compounds on the internal RFP control in the cell-based dual-reporter assay. (a) Ribavirin, GS-441524, and molnupiravir were included as reference RdRp inhibitors, while lycorine and lycorine hydrochloride were evaluated as candidate alkaloids. Cells co-transfected with the dual-reporter and FIPV RdRp-complex plasmids and treated with 0.01% DMSO served as the positive control, whereas cells transfected with the dual-reporter plasmid alone served as the negative control. Bright-field images showing cell morphology following treatment with each compound at the highest concentration tested are also presented. Scale bars, 200 μm for fluorescence images and 100 μm for bright-field images. (b) RFP fluorescence images showing the preserved internal control following treatment with lycorine hydrochloride at lower concentrations. Scale bar, 100 μm. (c) RFP fluorescence counts; numbers above each bar indicate the GFP/RFP ratio for the corresponding treatment. Data are presented as mean ± SD.

Table S1. Oligonucleotides for FIPV RdRp expression plasmids construction and viral quantification assay

Oligonucleotides for FIPV RdRp expression plasmids construction	
Primer name	Sequences 5′-3′
nsp7_F1	CGGCGGCAGCTCTAAACTTACAGAGATG
nsp7_R1	AAAACTCGAGTTACTGTAAAATAGTGGT
nsp8_F1	CGGCGGCAGCAGTGTAGCATCTGCT
nsp8_R1	AAAACTCGAGTTACTGCAACTTTGTGGT
nsp12_F1	CGGCGGCAGCTCAGAGTTATTTAGAGTG
nsp12_R1	AAAACTCGAGTTATTGCAAAACAGTGGA
**Oligonucleotides for viral quantification assay**	
Primer name	Sequences 5′-3′
FCoV 3′UTR-F [1–4]	GGCAACCCGATGTTTAAAACTGG
FCoV 3′UTR-R [1–4]	CACTAGATCCAG ACGTTAGCTC

The designated restriction site (*Xho*I) is underlined.

References

1. Herrewegh, A. A.; de Groot, R. J.; Cepica, A.; Egberink, H. F.; Horzinek, M. C.; Rottier, P. J. Detection of Feline Coronavirus RNA in Feces, Tissues, and Body Fluids of Naturally Infected Cats by Reverse Transcriptase PCR. *J. Clin. Microbiol.* 1995, *33* (3), 684–689. https://doi.org/10.1128/jcm.33.3.684-689.1995.
2. Theerawatanasirikul, S.; Kuo, C. J.; Phetcharat, N.; Lekcharoensuk, P. In Silico and in Vitro Analysis of Small Molecules and Natural Compounds Targeting the 3CL Protease of Feline Infectious Peritonitis Virus. *Antiviral Res.* 2020, *174*, 104697. https://doi.org/10.1016/j.antiviral.2019.104697.
3. Lueangaramkul, V., Mana, N., Thangthamniyom, N., Semkum, P., Lekcharoensuk, P., Limprasutr, V., Theerawatanasirikul, S. Repurposing FDA-Approved Drugs as Potential Inhibitors of Feline Infectious Peritonitis Virus 3CLpro: An Integrated In Silico and In Vitro Study with Synergistic Combination Analysis. *ACS Pharmacol. Transl. Sci.* 2026 *9*(6), 1451–1464. https://doi.org/10.1021/acsptsci.6c00016
4. Triratapiban, C.; Lueangaramkul, V.; Phecharat, N.; Pantanam, A.; Lekcharoensuk, P.; Theerawatanasirikul, S. First Study on in Vitro Antiviral and Virucidal Effects of Flavonoids against Feline Infectious Peritonitis Virus at the Early Stage of Infection. *Vet. World* 2023, *16* (3), 618–630. https://doi.org/10.14202/vetworld.2023.618-630.
